# ERCP-Net: a channel extension residual structure and adaptive channel attention mechanism for plant leaf disease classification network

**DOI:** 10.1038/s41598-024-54287-3

**Published:** 2024-02-20

**Authors:** Xiu Ma, Wei Chen, Yannan Xu

**Affiliations:** 1 Co-Innovation Center for the Sustainable Forestry in Southern China, Nanjing Forestry University, Nanjing, 210037 China; 2 East China Academy of Inventory and Planning of National Forestry and Grassland Administration, Hangzhou, 310019 China

**Keywords:** Image processing, Machine learning, Computer science

## Abstract

Plant leaf diseases are a major cause of plant mortality, especially in crops. Timely and accurately identifying disease types and implementing proper treatment measures in the early stages of leaf diseases are crucial for healthy plant growth. Traditional plant disease identification methods rely heavily on visual inspection by experts in plant pathology, which is time-consuming and requires a high level of expertise. So, this approach fails to gain widespread adoption. To overcome these challenges, we propose a channel extension residual structure and adaptive channel attention mechanism for plant leaf disease classification network (ERCP-Net). It consists of channel extension residual block (CER-Block), adaptive channel attention block (ACA-Block), and bidirectional information fusion block (BIF-Block). Meanwhile, an application for the real-time detection of plant leaf diseases is being created to assist precision agriculture in practical situations. Finally, experiments were conducted to compare our model with other state-of-the-art deep learning methods on the PlantVillage and AI Challenger 2018 datasets. Experimental results show that our model achieved an accuracy of 99.82% and 86.21%, respectively. Also, it demonstrates excellent robustness and scalability, highlighting its potential for practical implementation.

## Introduction

Plant leaf diseases decrease the efficiency of photosynthesis and seriously hinder the synthesis of organic matter and energy acquisition. It has become one of the main obstacles to achieving high yield and quality of crops. Meanwhile, various degrees of diseases impair the synthesis of nutrient proteins and result in yield reduction, reducing the economic efficiency of crops^1^. Traditional plant leaf disease identification mainly relies on the experience accumulated by generations of researchers in the plant production process, which requires a high level of professional knowledge for plant producers. However, discriminating plant leaf diseases by eyes has high subjectivity and is prone to errors, thus hindering the timely treatment of plants^2,3^. Therefore, for today’s agricultural production, it is necessary to develop a new system to liberate producers from the inefficient and complex process of plant leaf disease identification. Due to artificial intelligence’s rapid development, image processing and deep learning techniques are becoming increasingly mature. The application of deep learning technology^4–11^ to identifying plant leaf diseases intelligently has become a prominent trend, which helps to overcome the defects of traditional methods to improve plant yields^12^.

Considering the above issues, a new plant disease classification network is proposed in this paper to improve identification accuracy and efficiency. Meanwhile, a plant leaf disease identification application (APP) is presented to assist in identifying plant leaf diseases, thereby maximizing yields and ensuring sustainable agricultural development. To extract discriminative features for leaf disease classification, three different neural network blocks-the bidirectional information fusion block (BIF-Block), the adaptive channel attention block (ACA-Block), and the channel expansion residual block (CER-Block)-are specifically employed. Among them, the CER-Block adopts three pooling windows of different sizes and a residual structure to expand the model’s receptive field and output channels while maintaining a lower computational burden. The ACA-Block introduces an adaptive size distribution function with a reverse Gaussian probability density function into the Convolutional Block Attention Module (CBAM)^13^, enabling the model to focus on critical regions and channels containing leaf disease information. The BIF-Block establishes a bidirectional information pipeline among multi-level features to extract fine-grained information between multi-level features, thereby improving the robustness and accuracy of the network.

The main contributions of this work are summarized as follows:

- A novel ERCP-Net is proposed based on deep learning techniques, which effectively combines CER-Block, ACA-Block, and BIF-Block to achieve automatic recognition of plant leaf diseases.
- A convenient plant leaf disease identification APP is developed, which is equipped with a trained model and supports photographing, uploading, identification, and information feedback of plant leaves in real scenarios.
- The experimental results indicate that the proposed ERCP-Net achieved recognition accuracy of 99.82% and 86.21%, outperforming other state-of-the-art methods.

The rest of this paper is outlined as follows: “Related work” discusses prior research on plant leaf disease recognition using deep learning techniques. “Dataset processing” presents the experimental dataset and introduces the methods used for increasing the sample size. “Method” provides a detailed description of the method proposed in this work. “Experimental results and analysis” introduces the experimental setup and environment, presents, compares, and analyzes the experimental results to validate the feasibility of the proposed method. “Conclusion” concludes the whole study and provides insights into future research directions.

## Related work

### Deep learning techniques applied to plant leaf disease identification

Although deep learning networks such as VGG^14^, ResNet^15^, DenseNet^16^, and Efficientnet^17^, perform well in traditional classification tasks, they are not suitable for plant leaf disease recognition. He et al.^4^ proposed an end-to-end bilinear residual structure that can extract finer-grained features on plant leaf spots. Brahimiet al.^5^ identified tomato leaf disease images by using a convolutional neural network (CNN) that is trained on a dataset containing 14828 images of tomato leaves infected with nine diseases. Soujanya et al.^6^ used deep learning techniques to classify plant diseases and proposed a method to reduce the number of parameters and computational cost by adding an inverse convolution layer to the traditional AlexNet. The method achieved the best accuracy of 96.50$\%$. Singh et al.^7^ developed a multilayer CNN for classifying mango anthracnose leaves, and the proposed model obtains higher classification accuracy for mango anthracnose than other methods. Hussain et al.^8^ constructed a deep learning framework based on optimal feature selection for identifying multiple classes of foliar diseases of cucumber. Akram et al.^18^ added a low-pass output to the Retinex model for dataset preprocessing to improve the detection of small targets. Moreover, a classification method was developed based on the deep convolutional neural network to classify leaf diseases of five plants, and it obtained an accuracy of 97.80$\%$ on the PlantVillage dataset. Chen et al.^19^ extended VGG by applying transfer learning to the Inception module for pre-training, and the method achieved an accuracy of above 91.83$\%$ on the public dataset and 92.00$\%$ for the classification prediction of rice plant leaf disease images in complex contexts. Wang et al.^20^ classified the images of the apple black spot PlantVillage dataset according to the degree of disease as healthy leaves, mild, moderate, and severe diseased leaves based on expert opinion. Meanwhile, the researchers compared four classification networks, including VGG16, VGG19, Inception-V3, and ResNet50, and they concluded that the fine-tuned VGG16 model performed best, with a classification accuracy of 90.40$\%$ on the test set of disease severity assessment. Chohan et al.^21^ developed a plant leaf disease classification model based on CNNs, and the accuracy of the proposed model on the test set was 98.30$\%$. Akshai et al.^22^ trained three CNNs, including VGG, ResNet, and DenseNet, on the PlantVillage dataset. The results showed that DenseNet performed the best on the test set with an accuracy of 98.27$\%$. Hassan et al.^23^ constructed a deep learning model using residual connectivity and deep separable convolution. This model achieved an accuracy of 99.39$\%$ on the PlantVillage dataset. Atila et al.^24^ designed a modified EfficientNet model, which obtained an accuracy of 99.97$\%$ on the test set of the PlantVillage dataset.

### Attention mechanism techniques applied to plant leaf disease identification

Limited by the local perceptual field problem of convolutional operations, traditional CNNs tend to obtain local optimal solutions, resulting in the loss of feature information. Therefore, attention mechanisms that can selectively focus on the feature information of interest have been widely studied. For the attention mechanism in CNN, visual attention is divided into channel attention and spatial attention by Niu et al.^25^. The most commonly used attention mechanisms are Squeeze-and-Excitation Network (SENet)^26^ and CBAM^27^. Alirezazadeh et al.^28^ embedded the improved CBAM into their model to achieve an accuracy of 86.89$\%$ on the test set of the public dataset DiaMOS^29^. Yang et al.^30^ proposed an attention mechanism with weighted feature information fusion for fine-grained classification of 37 types of plant leaf diseases. The proposed attention mechanism combined with transfer learning achieved an accuracy of 95.62$\%$ on the test set. Zhao et al.^31^ incorporated an improved CBAM into ResNet to reduce redundant information extracted from the convolutional layer. The proposed model achieved an accuracy of 97.59$\%$ on a dataset of 16 tomato leaf diseases. Zhao et al.^32^ enhanced the channel attention in the CBAM structure by replacing the Shared MLP (Multilayer Perceptron) with two one-dimensional convolutions and modifying the kernel size of the one-dimensional convolution based on prior knowledge. For a dataset containing images of corn, potatoes, and tomatoes from the PlantVillage dataset, the model obtained an accuracy of 99.55$\%$ on the test set. Based on the analysis of the existing research, this work proposes to use the residual and attention mechanism to further improve the classification of plant leaf diseases. Table 1 summarizes the relevant research on the PlantVillage dataset.

**Table 1:** Comparison of different obfuscations in terms of their transformation capabilities.

Reference	Methods	Accuracy (%)
Akram et al.^18^	CNN	97.80
Wang et al.^20^	Proposed VGG16	90.40
Chohan et al.^21^	CNN	98.30
Akshai et al.^22^	DenseNet	98.27
Hassan et al.^23^	CNN	99.39
Atila et al.^24^	Proposed EfficientNet	99.97
Zhao et al.^32^	Proposed model	99.55

## Dataset processing

**Figure 1:**
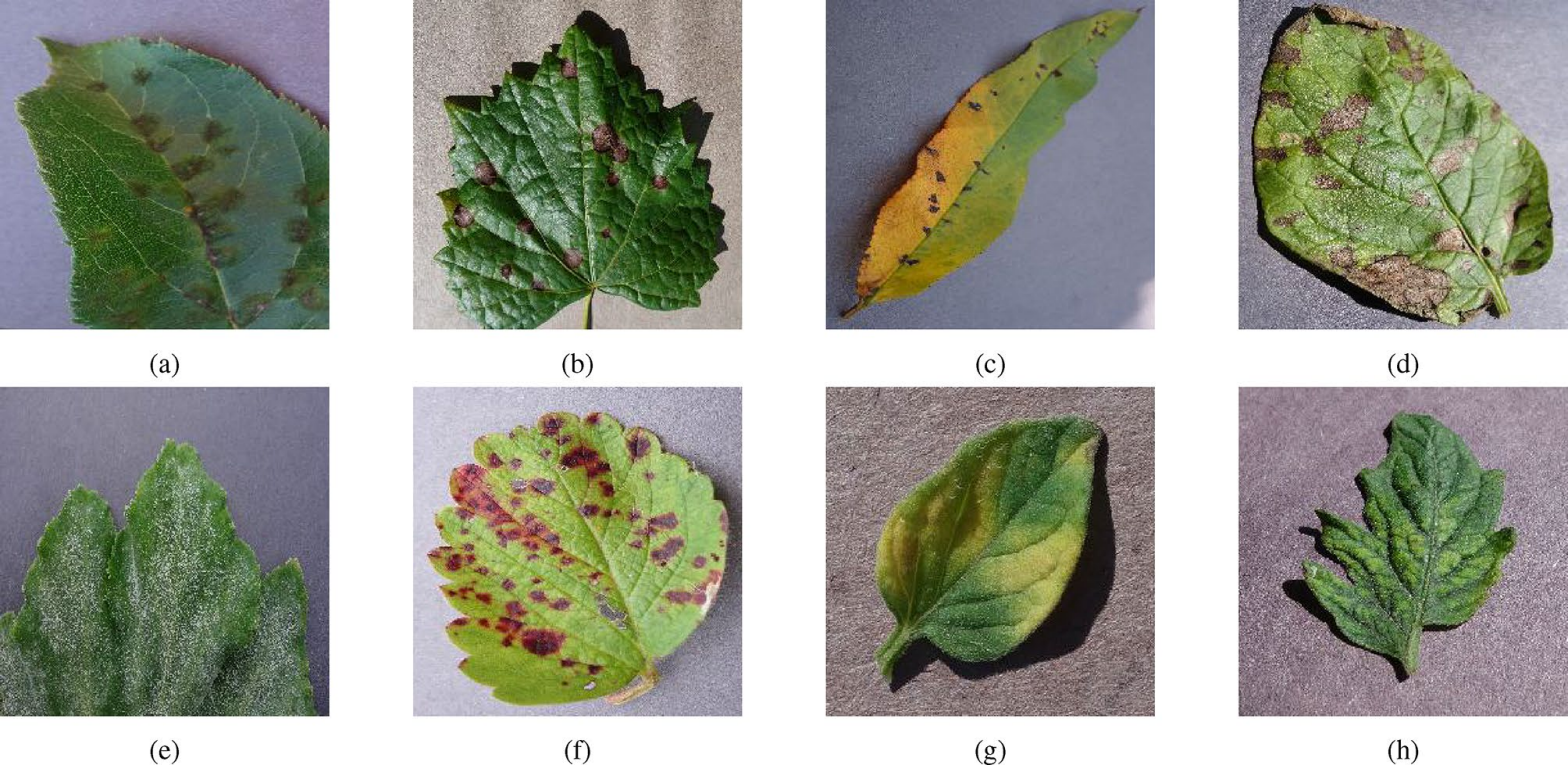
The sample results of plant leaf diseases: (**a**) apple scab, (**b**) grape black rot, (**c**) peach bacterial spot, (**d**) potato early blight, (**e**) squash powdery mildew, (**f**) strawberry leaf scorch, (**g**) tomato leaf mold, and (**h**) tomato mosaic virus.

The PlantVillage^33^ dataset used in this study is publicly available and authoritative. The PlantVillage does not represent real scenarios but lab conditions. This dataset includes leaf diseases of 14 types of plants with a total of 38 disease types. There are 54305 sample images in the dataset, and each image has three channels of R, G, and B. Fig. 1 shows some plant leaf disease images in the dataset.

To improve the generalization and robustness of the model, this work adopts four methods for data enhancement, including random horizontal flip, random vertical flip, random rotation of the image angle between 0 and 35 degrees, and the addition of Gaussian noise. The enhanced dataset has 60371 sample images in total. The dataset was divided into a training set, a validation set, and a test set at a ratio of 6:2:2.

In order to verify the performance of the model. There are 50,000 labeled images of crop leaves in the dataset used in the AI Challenger 2018. Ten plant species-apples, cherries, grapes, citrus, peaches, strawberries, tomatoes, peppers, maize, and potatoes-as well as twenty-seven distinct illnesses are depicted in the pictures. With 61 categories in all, the data collection offers rich and varied samples for researching illnesses and pests.

## Method

This section introduces our proposed method and its variants in detail. First, the CER-Block is presented, which can extract image information accurately by combining the ideas of channel expansion and residuals. Based on the CER-Block, the ER-Net is constructed, which is a backbone network for plant leaf disease classification. Second, the ACA-Block is designed, which makes the backbone network focus more on leaf disease information to reduce redundant information interference. Also, as shown in Fig. 5, the ACA-Block is embedded into the backbone network to build a stronger ERC-Net. Finally, the BIF-Block is proposed to improve the classification results’ robustness. Finally, the state-of-the-art classification model ERCP-Net is established, as shown in Fig. 6.

**Figure 2:**
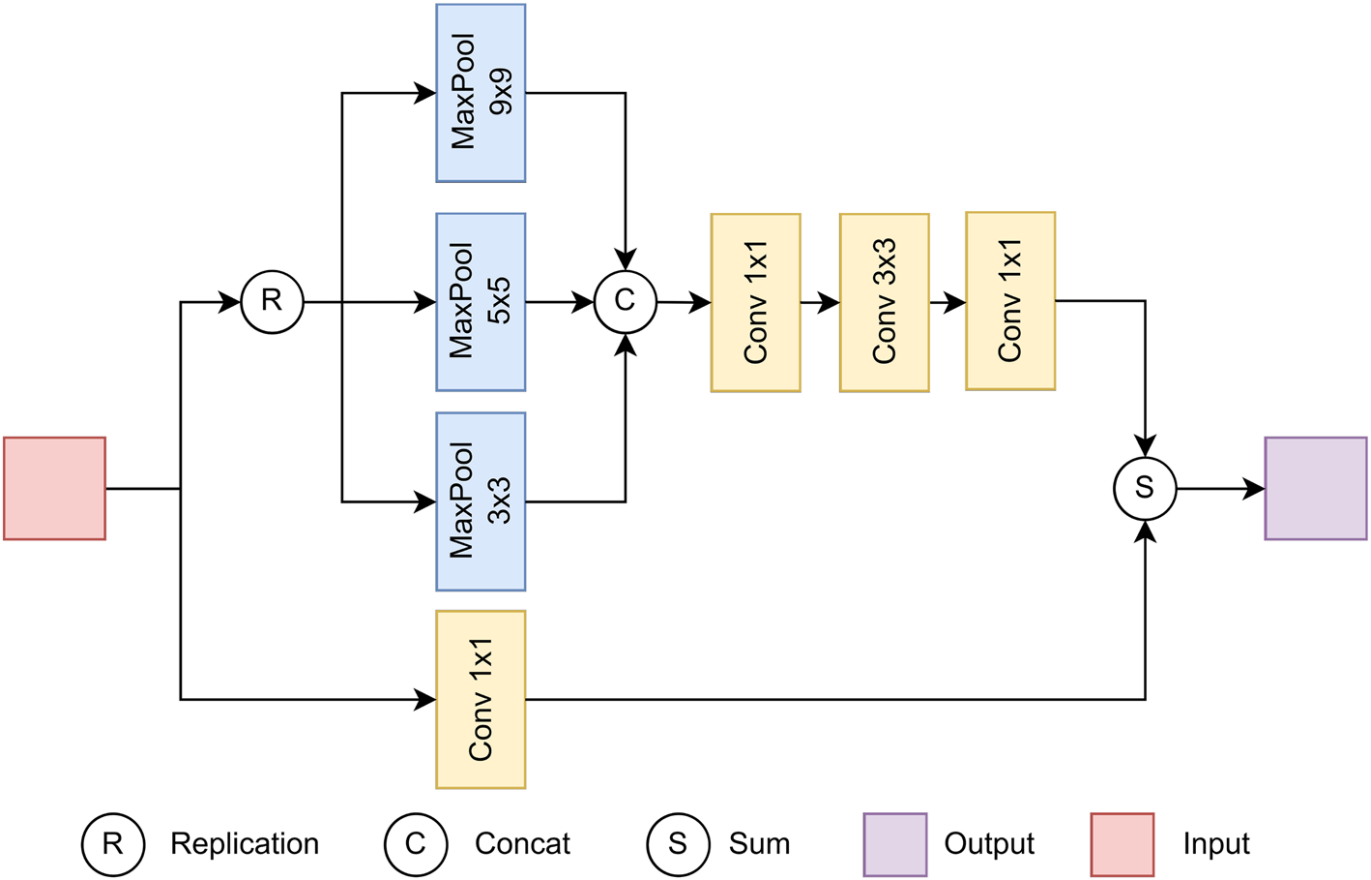
The structure of the CER-Block. “a$\times$b” means window size. Note that the stride of each component defaults to 1.

**Figure 3:**
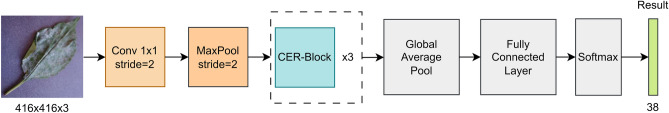
The framework of the ER-Net network.

### CER-Block and ER-Net

Traditional image classification networks usually use convolutional operations for channels to scale, which can increase the number of parameters. As the network deepens, numerous training parameters will incur a large computational burden and cause gradient information disappearance. To solve this problem, this paper proposes the channel expansion residual structure (CER-Block).

The CER-Block consists of two components: an image feature information extraction layer and a residual connection layer. The image feature information extraction layer consists of three max-pooling layers with different window sizes (3$\times$3, 5$\times$5, and 9$\times$9) and an information aggregation layer. This helps to expand the perceptual field while triple-expanding the number of channels without increasing the number of parameters. Then, the features obtained by max-pooling are fed to the information aggregation layer to make the network focus on leaf disease information from multiple perspectives. The information aggregation layer consists of three convolutions of different kernel sizes, i.e., 1, 3, and 1. The role of the convolution layer is to perform more abstract information aggregation from features.

Moreover, the residual connection layer comprises a convolution with a kernel size of 1, and this layer is essentially an additive node. It combines the gradient information of the upper layer with the output information of the first part while preserving the original state of the gradient. During the gradient information propagation, the risk of gradient explosion or gradient disappearance in the network is reduced Fig. 2 illustrates the structure of the CER-Block.

The backbone network ER-Net is constructed based on CER-Block. As shown in Fig. 3, the CER-Net comprises two down-sampling layers and three CER-Blocks. First, ER-Net receives the input images with a size of $416\times 416$ pixels. Then, the image is fed to a 7$\times$7 convolution layer with a stride of 2 and a max-pooling layer with a stride of 2. In this way, meaningless spatial information is suppressed, and discriminative channel information is improved. Since plant leaf diseases are often represented as composite features such as color, texture, and shape, it is difficult for a simple convolutional layer to transform composite feature information from simple to abstract. Therefore, the feature map is fed to three cascaded CER-Blocks to learn different and complementary plant leaf disease information from the feature map, thereby enhancing the network’s disease recognition capability. Finally, the abstract feature information is input to the prediction layer to obtain the final classification results.

**Figure 4:**
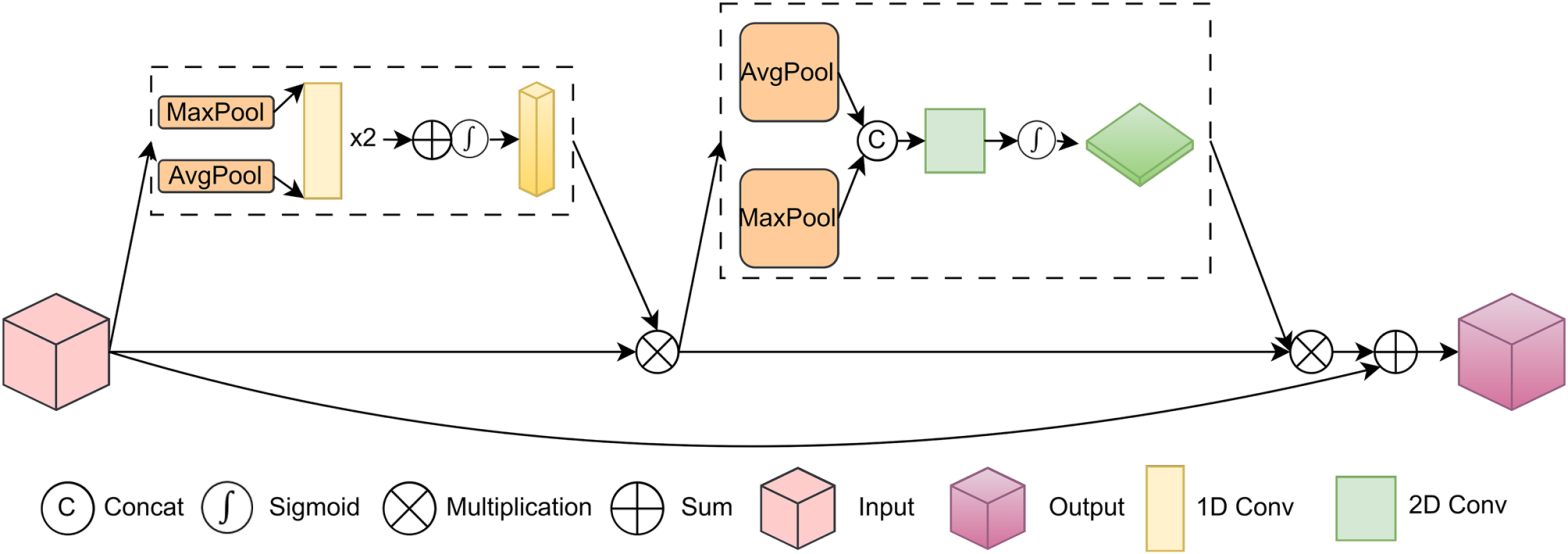
The structure of the ACA-Block. Note that the kernel size of 1D convolution is determined by an inverse Gaussian probability density function, and the kernel size of 2D convolution is set to 7$\times$7.

### ACA-Block and ERC-Net

CBAM^13^ is a classical attention mechanism module that combines channel and spatial attention. This module can be easily embedded into the backbone network for image classification to obtain better results. Zhao et al.^32^ proposed an improved channel attention module based on CBAM by modifying the shared MLP in the original channel attention module into two 1D convolutions and manually setting the kernel size of the 1D convolution. Then, they conducted a mass variant experiment about the convolution kernel size to achieve the best performance. Since the manual setting of kernel size is time-consuming, subjective, and random, this work uses the inverse Gaussian probability density function to project the kernel size of the 1D convolution adaptively.

Let the kernel size of the 1D convolution be *x*, and the number of channels of the feature map is *y*. Then, the original Gaussian probability density function is represented as:

1 $$\begin{aligned} y = \frac{1}{\sigma \sqrt{2 \pi }}e^{-\frac{(x-\mu )^2}{2\sigma ^2}} \end{aligned}$$

where $\mu$ is the mean and $\sigma$ is the variance. Moreover, the inverse Gaussian probability density function is as follows:

2 $$\begin{aligned} x = \sqrt{-2\sigma ^2\ln {(y\sigma \sqrt{2\pi })}} + \mu \end{aligned}$$

where $\mu$ represents the mean, and $\sigma$ represents the variance. First, the inverse Gaussian probability density function is applied to the channel attention module. Then, a residual connection is introduced to combine the complete gradient information of the CER-Block with the output information of the attention module while preserving the original state of the gradient. Based on this, the ACA-Block is constructed. Note that *x* is an integer greater than or equal to 1. Fig. 4 presents the framework of the proposed ACA-Block.

Compared with the CBAM and BAM, our ACA-Block has three improvements. First, we use two 1D convolutions in the channel attention module to replace the original 2D convolution. The two one-dimensional convolutions are not downsampled, which can better prevent information loss in the feature map. Second, the size of the convolution kernel in the two one-dimensional convolutions is calculated by the inverse Gaussian probability density function (IGPDF). It is an adaptive size distribution function that can change with the size of the feature maps, making the feature maps have a stronger correlation after convolution. Third, we add a new residual structure. This structure ensures that the input gradient information can retain its original state after ACA-Block. A comparison of the formulae for calculating the feature map information for the CBAM, BAM, and ACA-Block is as follows:

3 $$\begin{aligned} F'_{BAM}= & {} BN(MLP(AvgPool(F))) + M_{S} (F) \end{aligned}$$

4 $$\begin{aligned} F'_{CBAM}= & {} \sigma (MLP(AvgPool(F)) + MLP(MaxPool(F))) + M_{S} (F) \end{aligned}$$

5 $$\begin{aligned} F'_{ACA-Block}= & {} F + \sigma (IGPDF(AvgPool(F)) + IGPDF(MaxPool(F))) + M_{S} (F) \end{aligned}$$

where $F'_{BAM}, F'_{CBAM}, F'_{ACA-Block}$ are the outputs of the BAM, the CBAM, and the ACA-Block, respectively. *BN* is batch-normalization. *MLP* is a multilayer perceptron consisting of two two-dimensional convolutions. $M_{S}(\cdot )$ is spatial attention. *F* is input information. *IGPDF* is the inverse Gaussian probability density function. *AvgPool* is average pooling. *MaxPool* is max pooling.

**Figure 5:**
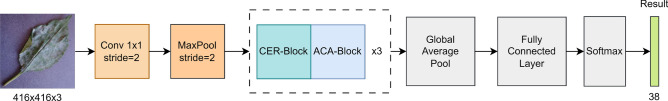
The framework of the ERC-Net network.

As illustrated in Fig. 5, the ACA-Block is embedded behind each CER-Block to make the backbone network focus more on the information of the leaf disease part to reduce the interference of redundant information. Based on this, the ERC-Net network is constructed. Since the number of channels of the feature maps obtained by the three CER-Blocks in the ERC-Net network differs, the kernel size of the 1D convolution calculated by the inverse Gaussian probability density function is also different. Because of this, the attention module ACA-Block receives feature maps of different scales and with different numbers of channels. From the above two aspects, the ACA-Block can filter out redundant information, focus on leaf disease features, and further enhance the information correlation between feature maps to improve the accuracy of plant leaf disease classification.

**Figure 6:**
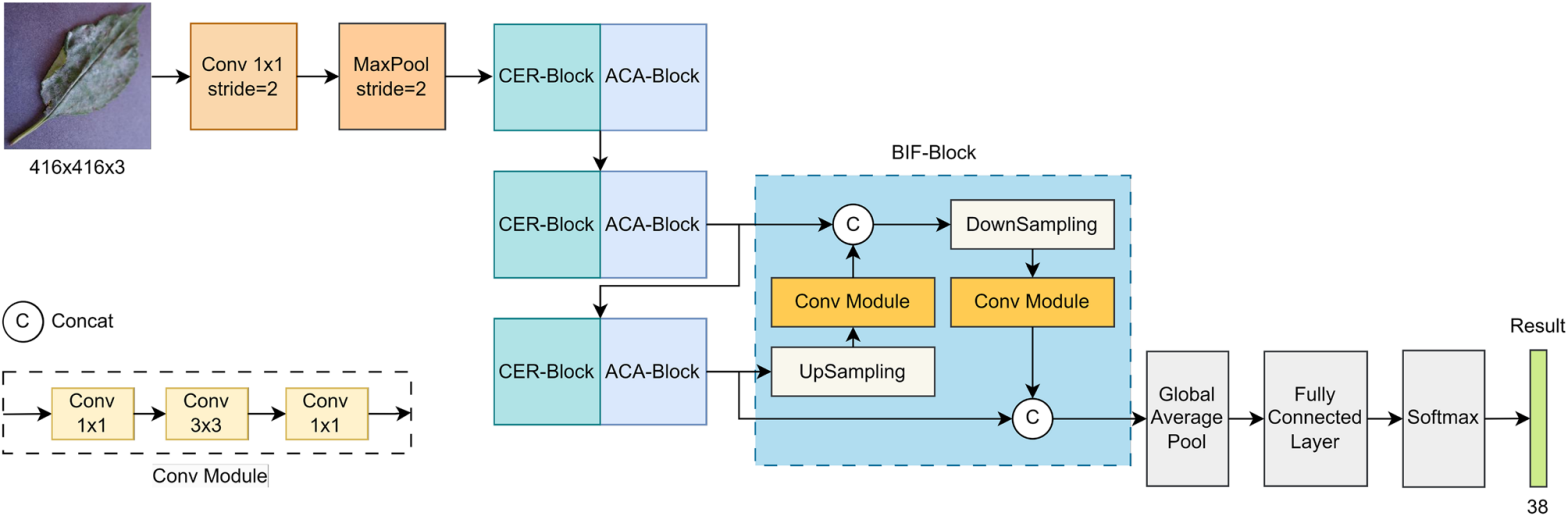
The framework of the ERCP-Net network.

### ERCP-Net

The traditional image classification network feeds the feature information extracted from top to bottom to the output layer to obtain the prediction results. Nevertheless, this output layer only focuses on the semantic information extracted from the deeper layers of the network. Meanwhile, it is difficult to focus on the pixel-level features of the image. To enable the classification network to focus on feature information from different aspects, this paper proposes a bidirectional information fusion block (BIF-Block) that incorporates feature map information from multiple perspectives. The improved output layer can focus on semantic and pixel-level information, thereby obtaining a robust prediction result. Fig. 6 illustrates the structure of the ERCP-Net network. Firstly, the traditional output layer of the ERC-Net network is removed. Secondly, the feature map information obtained from the third CER-Block+ACA-Block structure is upsampled and merged with the feature map information obtained from the second CER-Block+ACA-Block structure. The number of channels of the merged feature map is increased, i.e., deeper pixel information is added to the shallow semantic information. Then, the merged feature information is downsampled and merged again with the feature information obtained from the third CER-Block+ACA-Block structure to further enrich the semantic and pixel information. Finally, the final feature information is feedback to the output layer to obtain robust classification results. The output tensor dimensions for each layer of ERCP-Net are detailed in Table 2.

**Table 2:** The details of ERCP-Net and the tensor sizes of each output layer.

Layer	Tensor size
Input	[3,416,416]
Conv	[16,208,208]
MaxPool	[16,104,104]
CER-Block_1	[48,52,52]
ACA-Block_1	[48,52,52]
CER-Block_2	[144,26,26]
ACA-Block_2	[144,26,26]
CER-Block_3	[432,13,13]
ACA-Block_3	[432,13,13]
BIF-Block	[1296,13,13]
Global average pool	[1296,1,1]
Fully connected layer	[1296]
Softmax	[38]

## Experimental results and analysis

### Experimental setup

The experiment is conducted on a personal computer equipped with 32G RAM and an Nvidia GeForce RTX 3060 graphics card with 12G video memory, and the computer runs the Ubuntu 18.04.6 LTS operating system.

**Table 3:** The training parameters about ERCP-Net.

Parameter	Value
Batch size	16
Init learning rate	0.01
Epochs	100
Optimizer	SGD (momentum = 0.9, weight_decay = le−4)
Loss function	Cross-entropy

The deep learning libraries are Pytorch 1.8.0 and Python 3.8.15. The training parameters are set as follows: The batch size is set to 16, and the initial learning rate is set to 0.01. The model is trained for 100 epochs using the SGD optimizer and the cross-entropy loss function. The accuracy on the validation set is monitored during training, and the learning rate is decreased when the accuracy does not increase for three epochs  (Eq. 6). The detailed setting of the training parameters is listed Table 3.

6 $$\begin{aligned} LR = 0.3 \times lr \end{aligned}$$

where *lr* and *LR* denote the learning rate of the previous epoch and the current epoch, respectively.

### Evaluation metrics

To verify the feasibility of our method, accuracy, precision and recall are taken as the evaluation index for the experiment. The value of this index is calculated based on true positives (TP), true negatives (TN), false positives (FP), and false negatives (FN). The calculation formula is given below.

7 $$\begin{aligned} Accuracy= & {} \frac{TP + TN}{TP + TN + FP + FN} \end{aligned}$$

8 $$\begin{aligned} Precision= & {} \frac{TP}{TP + FP} \end{aligned}$$

9 $$\begin{aligned} Recall= & {} \frac{TP}{TP + FN} \end{aligned}$$

### Comparison and analysis

In this section, the performance differences between the proposed method and other state-of-the-art methods are compared to demonstrate the superiority of ERCP-Net in leaf disease spot recognition. Table 4 shows the accuracy of ERCP-Net and ten popular methods on the PlantVillage dataset. The results indicate that ERCP-Net achieves the best accuracy of 99.82%, surpassing the second-place by 0.27%. Although our result is lower than the first place, it may be related to the way the dataset is divided and enhanced. Meanwhile, the experimental results demonstrate that ERCP-Net performs better in leaf disease spot recognition than classical image classification networks, including VGG19, Inception-V3, DenseNet, EfficientNet, and ResNet50. On the PlantVillage dataset, Vo et al.^34^ also achieved an accuracy of 99.77% by combining EfficientNetB0 with MobileNetV2, Wang et al.^35^ achieved a 99.77% accuracy rate using the proposed methodology. Zhao et al.^32^ improved the CBAM structure of the channel attention by manually modifying the kernel size of the 1D convolution. The classification accuracy of the proposed model on the PlantVillage dataset is 99.55$\%$. The performance of our suggested model on AI challenger 2018 is displayed in Table 5. As we can see, our model’s accuracy of 86.21% outperforms the widely used pest and disease classification methods now in use. Compared to these methods, our method performs the best. The experimental results indicate that using the inverse Gaussian probability density function to adaptively adjust the convolution kernel size is reliable and advantageous.

**Table 4:** Comparison of ERCP-Net and state-of-the-art methods on the PlantVillage test set.

Reference	Methods	Accuracy (%)
Wang et al.^20^	Proposed VGG16	90.40
Khan et al.^18^	CNN	97.80
Akshai et al.^22^	DenseNet	98.27
Chohan et al.^21^	CNN	98.30
Kaushik et al.^36^	CNN	97.10
Hassan et al.^23^	CNN	99.39
Zhang et al.^37^	CNN	99.40
Atila et al.^24^	Proposed EfficientNet	**99.97**
Sutaji et al.^38^	CNN	99.52
Zhao et al.^32^	Proposed model	99.55
Vo et al.^34^	EffcientNetB0+MobileNetV2	99.77
Wang et al.^35^	ResNet101+Attention	99.82
Ours	ERCP-Net	99.82

The highest performance is marked in bold.

**Table 5:** Performance comparison among different work on the AI challenger 2018 dataset

Reference	Year	Network	Accuracy (%)
Ferentinos^39^	2018	VGG	82.57
Too et al.^17^	2019	DenseNet	84.25
Kamal et al.^40^	2019	MobileNet	83.74
Chen et al.^19^	2020	VGG19 variant	83.61
Ramamurthy et al.^41^	2020	CNN+attnetion	78.12
Ronghua Gao et al.^42^	2021	ResNet18 variant +attention	86.09
Zhao et al.^32^	2022	CNN+attention	84.91
Li et al.^43^	2023	CNN	85.73
Singh Thakur et al.^44^	2023	VGG variant	85.37
Ours	2023	ERCP-Net	**86.21**

The best performance is shown in bold.

**Table 6:** Comparison of model performance on the AI Challenger 2018 and PlantVillage datasets.

#	Model	AI Challenger 2018			PlantVillage		
		Accuracy (%)	Avg precision (%)	Avg recall (%)	Accuracy (%)	Avg precision (%)	Avg recall (%)
1	ER-Net	83.51	81.56	81.95	99.74	99.65	99.68
2	ER-Net-inceptionA	83.00	80.55	80.10	99.70	99.61	99.66
3	ER-Net+CBAM	82.86	80.23	79.87	99.69	99.60	99.66
4	ERC-Net	85.12	83.26	82.12	99.76	99.69	99.71
5	ERCP-Net	86.21	84.12	83.94	99.82	99.78	99.81

### Ablation and analysis

To assess the effectiveness of the proposed modules and conduct a thorough analysis of our ERCP-Net, we performed ablation studies on two datasets: PlantVillage^33^ and AI Challenger 2018.

Table 6 presents the performance of five model variants: ER-Net (#1), ER-Net-inceptionA (#2), ER-Net+CBAM (#3), ERC-Net (#4), and ERCP-Net (#5). To expand the channel number using deep learning techniques without increasing the parameter count, we propose the CER-Block. Utilizing this block, we first constructed the ER-Net (#1), a leaf disease spot classification network. Thanks to the CER-Block, ER-Net achieved accuracies of 99.74% and 83.51$\%$ on the PlantVillage and AI Challenger 2018 benchmarks, respectively. Furthermore, we replaced the CER-Block with the Inception-A block from Inception v4^45^ under identical experimental conditions. The introduction of the Inception-A block resulted in a decrease in accuracy, attributed to its inability to capture large-scale information and the loss of discriminative information through residual connections via the pooling layer. Then, the original CBAM^13^ module is embedded into the ER-Net, and the accuracy of the model decreases to 99.69$\%$ and 82.86$\%$, respectively. This is because some plant leaf diseases are affected by composite features such as color, texture, and shape, leading to a lower fit between the original CBAM and the ER-Net network. Subsequently, by embedding the original CBAM^13^ module into ER-Net, we observed a decrease in accuracy to 99.69% and 82.86% on the respective datasets. This decline is likely due to the inability of the original CBAM to adequately account for the composite features, such as color, texture, and shape, that characterize some plant leaf diseases, resulting in a suboptimal fit with the ER-Net network. To enhance the compatibility between CBAM and ER-Net, we improved the original CBAM and proposed the ACA-Block. Incorporating the ACA-Block, we developed ERC-Net, the second leaf disease spot classification network. ERC-Net showed improved accuracies of 99.76% and 85.12% on the PlantVillage and AI Challenger 2018 datasets, respectively. Additionally, ERC-Net demonstrated increased average precision and average recall rates of 83.26% and 82.12%, correspondingly, on the AI Challenger 2018 benchmark. Ultimately, by replacing the traditional output layer with the BIF-Block, we designed ERCP-Net. Leveraging the BIF-Block allows ERCP-Net to integrate multi-perspective feature information, focusing on both semantic and pixel-level details. Experimental results reveal that ERCP-Net outperforms all previous models, achieving accuracies of 99.82% and 86.21%, average precisions of 99.78% and 84.12%, and average recalls of 99.81% and 83.94% on the PlantVillage and AI Challenger 2018 benchmarks, respectively. These findings underscore ERCP-Net’s superior capability in addressing complex image recognition tasks across diverse datasets.

**Figure 7:**
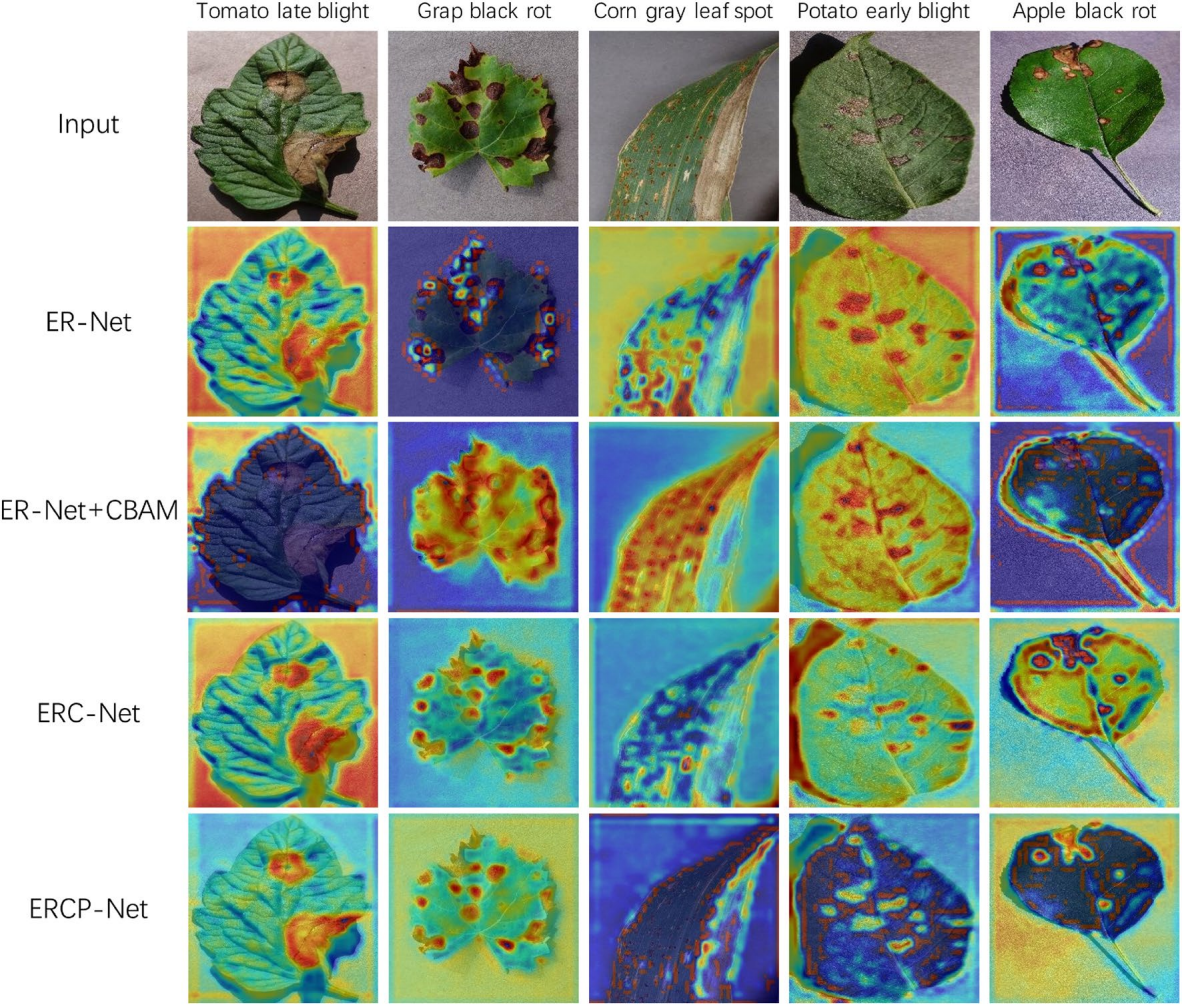
The visualization of the heatmap of the four variants. The sampled leaf disease images consist of tomato late blight, grape black rot, corn gray leaf spot, potato early blight, and apple black rot.

### Visual analysis

#### Heatmap

To further investigate the impact of each module, the heatmap was used to present the attention regions of each model. The algorithm used for the heatmap is Grad-CAM^46^. Grad-CAM decodes the importance of each feature map for a specific class by analyzing the gradient in the convolutional layer. As shown in Fig. 7, five types of leaf disease images are taken as input to show the heatmap of the four models. The ER-Net is our constructed base leaf disease classification model based on CER-Block. As shown in the second row, it can identify the diseased regions in the leaf, enabling leaf disease classification. In the third row, the ER-Net incorporates the attention module CBAM^13^, which is designed for classic classification networks. Obviously, the CBAM is not suitable for leaf disease classification tasks. After the introduction of CBAM, the attention region of the model is confused, leading to unreliable classification results. In the fourth row, the CBAM is replaced with the proposed ACA-Block to form the ERC-Net. It can be observed that ERC-Net focuses on leaf disease regions but fails to learn fine-grained information in the images. In the last row, the BIF-Block is applied to fuse multi-perspective information and obtain the proposed ERCP-Net. The ERCP-Net can accurately and comprehensively focus on the disease regions in the leaf, achieving the best classification performance.

#### Confusion matrix

**Figure 8:**
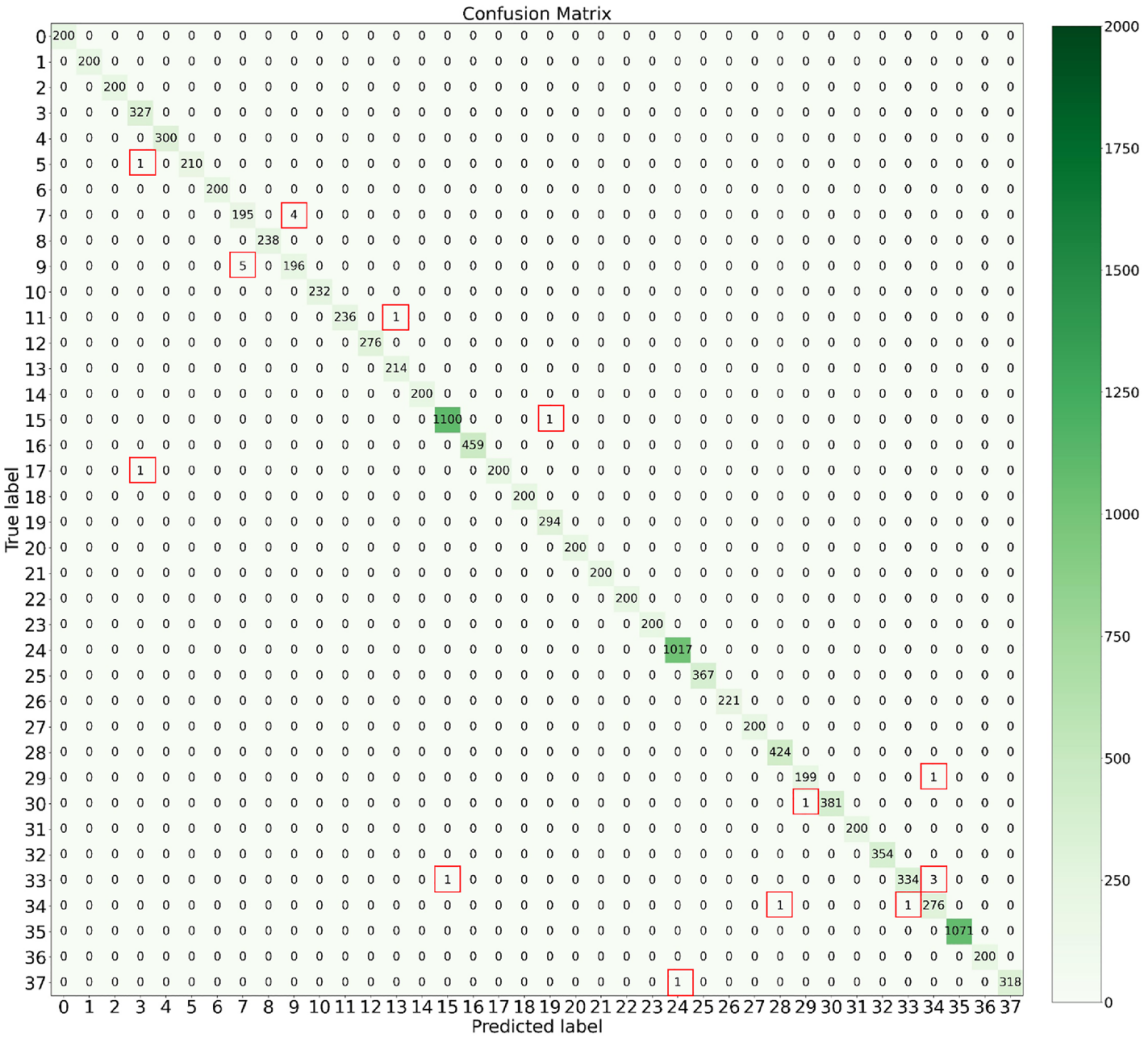
The confusion matrix of the classification results of ERCP-Net. The x and y axes in the confusion matrix correspond to the 38 categories of IDs.

To identify the weaknesses of ERCP-Net, the confusion matrix is plotted in Fig. 8. It shows that our ERCP-Net has difficulty in distinguishing between the categories ”Corn gray leaf spot” and ”Corn northern leaf blight”, ”Tomato two spider mite”, and ”Tomato target spot”. Meanwhile, there exists an issue of uneven data distribution in the PlantVillage data. It is speculated that the deficiency in focusing on hard samples might be due to the cross-entropy loss. In future work, we will explore the potential enhancement by using the focal loss^47^.

**Figure 9:**
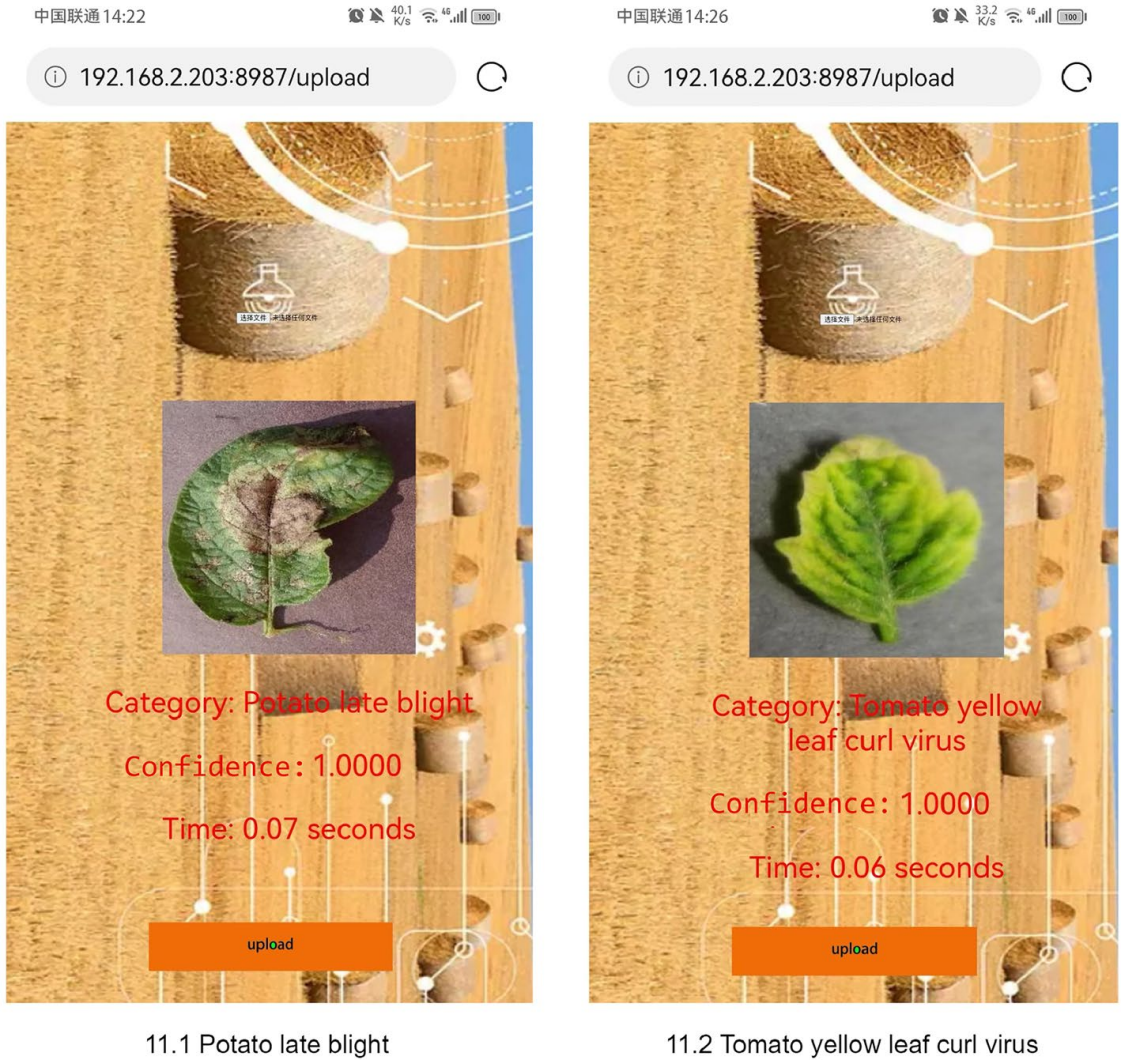
The results of plant leaf disease detection by the developed APP.

### Plant leaf disease identification APP

Currently, few plant leaf diseases can be identified with lightweight smart devices. An APP for plant leaf disease identification is built to make the study of plant leaf diseases more convenient and common. The creation of an APP involves three processes. We should first define scope and target. The App is a portable application made to assist farmers in promptly identifying the type of leaf disease and promptly implementing preventive measures. Second, the APP interface presents information in an understandable manner, taking into account both design and user experience. The information display box and the picture upload button are the two components of the interface. When users launch the APP, they may quickly learn how to use it and its function. Thirdly, the front and back end comprise an application. Python is used for front end development, and Flask is the framework. Python is used for back end development, while Pytorch is used as a framework. A LAN must contain both the front end and the back end. The APP’s recognition algorithm is based on the ERCP-Net algorithm. The APP consists of two main functions: uploading pictures and recognizing plant leaf disease. The second function depends on the trained ERCP-Net model, which has an accuracy of 99.82$\%$ on the test set. When the images are uploaded, the terminal invokes our algorithm to recognize the images and display the results on the main screen. Specifically, three results are displayed: the type of plant leaf disease, recognition confidence, and inference time (in seconds). The APP’s performance has been tested extensively, and some results are shown in Fig. 9. Fig. 9 shows the results of the APP for identifying potato late blight, where the identification category is also potato late blight, with an confidence of 100$\%$ and inference time of 0.07s. The experimental results show that the APP equipped with the ERCP-Net model can identify plant leaf diseases easily and in real-time with high confidence and speed. It can prevent the spread of diseases and ensure the healthy growth of plants, assisting with precision agriculture applications.

**Figure 10:**
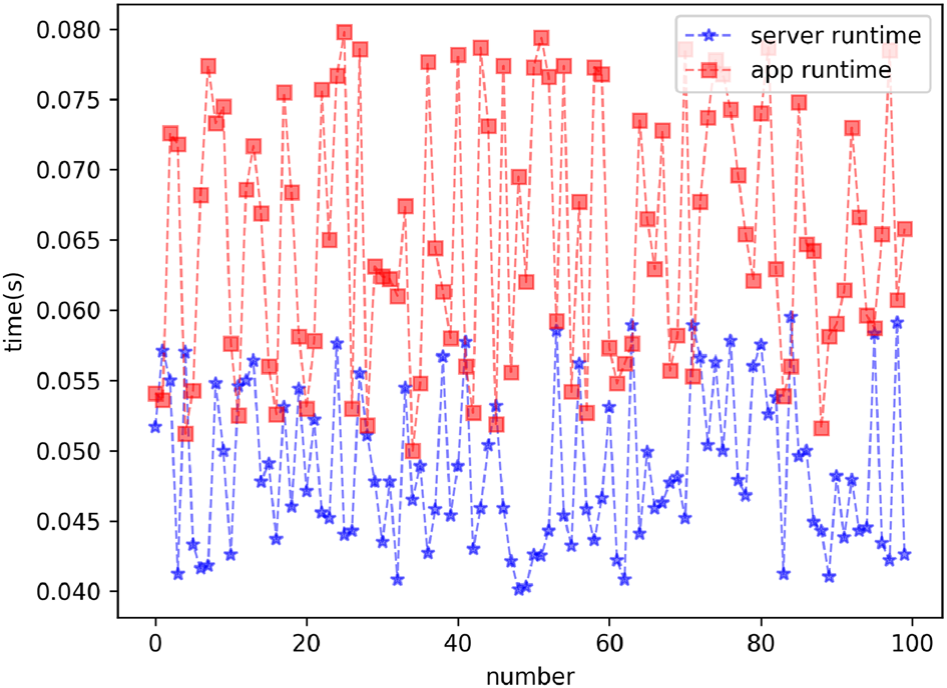
Server runtime and app runtime.

### Runtime

In order to show the real-time performance of the model more clearly, we conducted experiments on two devices. The first device is our experimental server. The specification of the machine is conducted on a personal computer equipped with 32G RAM and an Nvidia GeForce RTX 3060 graphics card with 12G video memory, and the computer runs the Ubuntu 18.04.6 LTS operating system. The second device is an Android phone with the APP on it. The specification of the machine is 16G RAM and Snapdragon 8 mobile platform Gen 2. The APP runs the Android 13. We performed 100 inference tests on two separate devices. The running time of the first device is from 0.04s to 0.06s. The average running time is 0.048s. The running time of the second device is from 0.05s to 0.08s. The average running time is 0.065s. Based on the experimental results, it can be seen that there is a difference of 0.017s in the average running time of the two devices, which is within the acceptable range. The reasons for the difference in the running time of the two devices may be the device specifications and network latency. Fig.10 illustrates the running time of the two devices.

### Limitation and future work

Despite the promising results achieved by our proposed ERCP-Net model, there are certain limitations that should be acknowledged. Firstly, our model’s performance might be influenced by variations in environmental conditions and imaging setups, as the dataset used for training and evaluation may not cover all possible scenarios. Additionally, the current version of ERCP-Net might face challenges in cases of extremely rare or unseen leaf diseases, as the training dataset may not comprehensively represent the entire spectrum of plant leaf diseases.

To address the aforementioned limitations and further enhance the applicability of our model, future research directions include expanding the dataset to encompass a wider range of environmental conditions, imaging angles, and disease manifestations. The introduction of transfer learning techniques, pre-training on diverse datasets, and fine-tuning on specific plant species could contribute to improved generalization. Moreover, incorporating real-time disease monitoring capabilities and deploying the model in field conditions would be crucial for practical applications. Future research should focus on investigating interpretability techniques to comprehend the model’s decision-making process and on user studies to evaluate the model’s performance in practical situations.

## Conclusion

In this paper, a new plant leaf disease classification network is developed based on deep learning and an attention mechanism. Firstly, based on multi-scale pooling and residual connection, the ER-Block is designed for image feature information extraction, which can triple the number of channels without increasing the number of network parameters while expanding the perceptual field and extracting feature information at multiple scales. Secondly, the ACA-Block is developed, which employs the inverse Gaussian probability density function to project the kernel size of the 1D convolution adaptively. In this way, it can receive feature maps of different scales and different numbers of channels, thereby making the backbone network focus more on the information of the leaf disease part and reducing the interference of redundant information. Finally, a feature fusion result prediction structure is proposed to improve the robustness of the network. Then, the plant leaf disease classification network ERCP-Net is constructed based on the above modules. ERCP-Net can reduce redundant information interference and focus more on leaf disease features by transforming the shallow image information into more abstract feature information. Also, unlike traditional image classification networks, ERCP-Net can focus on semantic and pixel-level information. Finally, an app is developed to identify plant leaf diseases with a simplified detection procedure. Experimental results show that the proposed ERCP-Net network performs better than existing approaches on the PlantVillage and AI challenger 2018 datasets, with accuracy of 99.82% and 86.21%.

In future research, we will conduct in-depth research on the following two aspects. First, we will introduce small-sample learning to recognize some small-sample disease categories effectively. Plant disease recognition relies on a large amount of plant leaf image data. Nevertheless, in actual production, it is a great challenge to obtain the expected results for recognizing some disease categories whose images are difficult to collect or label. Second, the proposed model will be deployed to more sophisticated and intelligent machines, such as agricultural mobile robots, to develop an intelligent integrated process for data processing, identification, and detection.

## Data Availability

This study did not report any data. The proposed method was evaluated on publicly available diseases and insect pests detection datasets widely used in object detection: PlantVillage (https://data.mendeley.com/datasets/tywbtsjrjv/1) and AI challenger 2018 (https://aistudio.baidu.com/datasetdetail/76075).
